# Scan-associated anxiety (scanxiety): the enigma of emotional breathing oscillations at 0.32 Hz (19 bpm)

**DOI:** 10.3389/fnins.2024.1384993

**Published:** 2024-04-04

**Authors:** Gert Pfurtscheller, Beate Rassler, Gerhard Schwarz, Wolfgang Klimesch

**Affiliations:** ^1^Institute of Neural Engineering, Graz University of Technology, Graz, Austria; ^2^Carl-Ludwig-Institute of Physiology, University of Leipzig, Leipzig, Germany; ^3^Department of Anaesthesiology and Intensive Care Medicine, Medical University of Graz, Graz, Austria; ^4^Centre of Cognitive Neuroscience, University of Salzburg, Salzburg, Austria

**Keywords:** MRI-related anxiety, emotional breathing, claustrophobia, nasal respiration, brain–body interaction, binary hierarchy model

## Abstract

MRI-related anxiety in healthy participants is often characterized by a dominant breathing frequency at around 0.32 Hz (19 breaths per minute, bpm) at the beginning but in a few cases also at the end of scanning. Breathing waves at 19 bpm are also observed in patients with anxiety independently of the scanned body part. In patients with medically intractable epilepsy and intracranial electroencephalography (iEEG), spontaneous breathing through the nose varied between 0.24 and 0.37 Hz (~19 bpm). Remarkable is the similarity of the observed breathing rates at around 0.32 Hz during different types of anxiety states (e.g., epilepsy, cancer, claustrophobia) with the preferred breathing frequency of 0.32 Hz (19 bpm), which is predicted by the binary hierarchy model of Klimesch. This elevated breathing frequency most likely reflects an emotional processing state, in which energy demands are minimized due to a harmonic coupling ratio with other brain–body oscillations.

## Introduction

Magnetic resonance imaging (MRI) examinations in patients have often been aborted due to claustrophobia or strong anxiety. The results of a meta-analysis (Munn et al., 2015) and a review of claustrophobia incidence in MRI (Hudson et al., 2022) have shown that about 1–2% of people who undergo MRI scanning of different body parts experienced a claustrophobic reaction. In about 1% of all examinations, the claustrophobic reaction has led to a premature termination of the scan. With about 1.3 million MRIs performed in Australia in 2020 (Homewood and Hewis, 2023), this amounts to around 13.000 aborted examinations. This is a high number causing high costs and delays of the diagnosis in patients that needs further intensive examination.

The recently introduced term scan-associated anxiety (“scanxiety”) was coined to describe anxiety or distress, which emerge when subjects have to undergo an imaging procedure (with, e.g., positron emission tomography, computed tomography, magnetic resonance imaging, or magnetic resonance mammography). There are at least two main sources that induce anxiety. One is related to the possible diagnosis of a serious disease, such as cancer, the other is related to the specific examination method, which in extreme cases can lead to claustrophobia or panic attacks (Harris et al., 2001). The importance of “scanxiety” is underlined in the reviews by Bui et al. (2021) and Derry-Vick et al. (2023). Scanxiety is heightened during a pre-scan period and during the time in which people have to wait for the results of the diagnosis.

In a study with 44 patients, who were MRI scanned at different locations (e.g., knee, shoulder, lumbar spine, cervical spine and head) and who were tested with the State–Trait Anxiety Inventory (STAI; Spielberger et al., 2009), three subjects (7%) were withdrawn from the study due to strong anxiety or an onset of a panic attack (Dziuda et al., 2019). While the anxiety score was evaluated before and after the scanning procedure, breathing rate (BR) was acquired by a fibre-optic sensory system for 2 min immediately after the begin of scanning and 2 min before the end of scanning. The anxiety score decreased from a mean value of 36.9 before to 34.4 after scanning, and the mean BR decreased significantly from 18.7 to 17.4 bpm. In summarizing, this study (Dziuda et al., 2019), showed that (i) abortion rate was around 7%, (ii) BR increased during scanxiety to about 19 bpm (0.32 Hz) on average and in some cases up to 30 bpm, (iii) anticipated scanxiety was relatively independent of the scanned body part, and (iv) there was no correlation between anxiety level tested with STAI and BR values.

This relatively high rate of MRI abortions needs special attention and further research. Therefore, a study in healthy subjects without any former MRI experience with scanning periods over a number of resting states was performed (Pfurtscheller et al., 2018). One important point of this study was a focus on the dynamics of the anxiety level with habituation effects, and the “switch-off” of respiratory sinus arrhythmia (RSA) also known as negative RSA (Rassler et al., 2018, 2023). Another point was the investigation of the information flow (directed coupling) between brain and body in the high frequency band (0.2–0.4 Hz) close to the enhanced BR of 19 bpm (corresponds to 0.32 Hz) reported in patients (Dziuda et al., 2019).

Anxiety and distress are not only a problem associated with scanning, but are also observed during intracranial electroencephalographic (iEEG) recordings in patients with medically intractable epilepsy. Remarkably, also in this situation with iEEG recording and breathing cycle control by piezoelectric manometer within a nasal cannula device (Zelano et al., 2016; Herrero et al., 2018), the breathing rate varied between 0.24–0.37 Hz close to 19 bpm.

## Autonomic and emotional breathing in humans

Neurons in the PreBötzinger complex, located in the brainstem, act as pacemaker for respiration (Menuet et al., 2020). Two different conditions should be distinguished, autonomic breathing during a relaxed resting situation, and “emotional” breathing when subjects are confronted with emotionally unpleasant situations (Homma and Masaoka, 2008). Whereas autonomic breathing rate (aBR) varies between about 0.20–0.25 Hz (12–15 bpm) (Ebert et al., 2000; Rassler, 2000; Rassler and Raabe, 2003; Fleming et al., 2011), the emotional breathing rate (eBR) is around 0.32 Hz (19 bpm).

A nice example for the differences between aBR and eBR can be found in a study by Kato et al. (2017). Healthy subjects were sitting on a chair, wearing a facemask connected to a respiratory monitor in a quiet room. Respiratory rate and their parameters were measured breath by breath, and the anxiety level was monitored by Spielberger’s STAI. The subjects were divided into two groups, one with lower state anxiety and aBR = 15.0 bpm as compared to a group with higher state anxiety and eBR = 17.2 bpm. The difference of the BR was significant (*p* < 0.01). This is a good example to demonstrate the susceptibility and power of BR to monitor anxiety or scanxiety.

## Problem-related comments on data processing

When studying scanxiety in patients and MRI-related anxiety in healthy people by analyzing cardiac RR intervals (RRI) and respiration, it is not sufficient to investigate brain-heart interactions (Silvani et al., 2016; Candia-Rivera et al., 2022) or cardiorespiratory coupling (Friedman et al., 2012; Abreu et al., 2023). What is additionally needed are blood oxygen level dependent (BOLD) data without standard preprocessing (global signal regression, independent components analysis and retrospective correction), as respiration-related neural activity is typically considered noise and often removed (Goheen et al., 2023). An interesting type of coupling, which supports the validity of the binary hierarchy model of Klimesch (2018) is cardioventilatory coupling (Elstad et al., 2018). This type of coupling refers to the number of heart beats within a breathing cycle. Quite often it is found that heart rate is fourfold the breathing rate (Tzeng et al., 2003). With respect to emotional breathing, this tight cardiorespiratory coupling may be considered an important mechanism of emotional control. BOLD, RRI and respiration signals can be studied by calculating the Directed Transfer Function (DTF), which is based on the Granger causality principle (Granger, 1969; Kaminski and Blinowska, 1991). Therefore, this approach was used in the studies by Pfurtscheller et al. (2022, 2023).

## Breathing rate in healthy participants with MRI-related anxiety

The results of a resting state study with repeated measurements of physiological signals (electrocardiogram, respiration, BOLD) within four resting states in 23 healthy participants have been published elsewhere (Pfurtscheller et al., 2018, 2020, 2021, 2022, 2023). The main findings were: (i) The majority of MRI-naïve participants showed the highest anxiety score (AS) in the first resting state with a subsequent decrease of anxiety. A few participants displayed an increase in anxiety at the end of the sessions similar to a study reported by Chapman et al. (2010). (ii) Participants with relatively high anxiety in the first but also in a few of the last resting states displayed a high percentage of emotional breathing oscillations around 0.32 Hz ± 0.03 (Rassler et al., 2023). In six subjects, eBR of about 0.32 Hz was dominant in 63–97% of the resting state epoch. (iii) Anxiety processing is not only associated with a slightly enhanced eBR of 0.32 Hz (19 bpm) but in some cases also with very slow breathing waves (BR ≤0.2 Hz). (iv) The measurement of the information flow revealed a strong upwards flow from pontine structures in the brainstem to the prefrontal cortex in the 0.1–0.2 Hz band centered at 0.15/0.16 Hz (Pfurtscheller et al., 2020, 2022) and a strong flow from cardiac to respiratory systems in the 0.2–0.4 Hz band (Pfurtscheller et al., 2023).

## Interaction between cardiac RR interval oscillations and breathing waves

**Figure 1:**
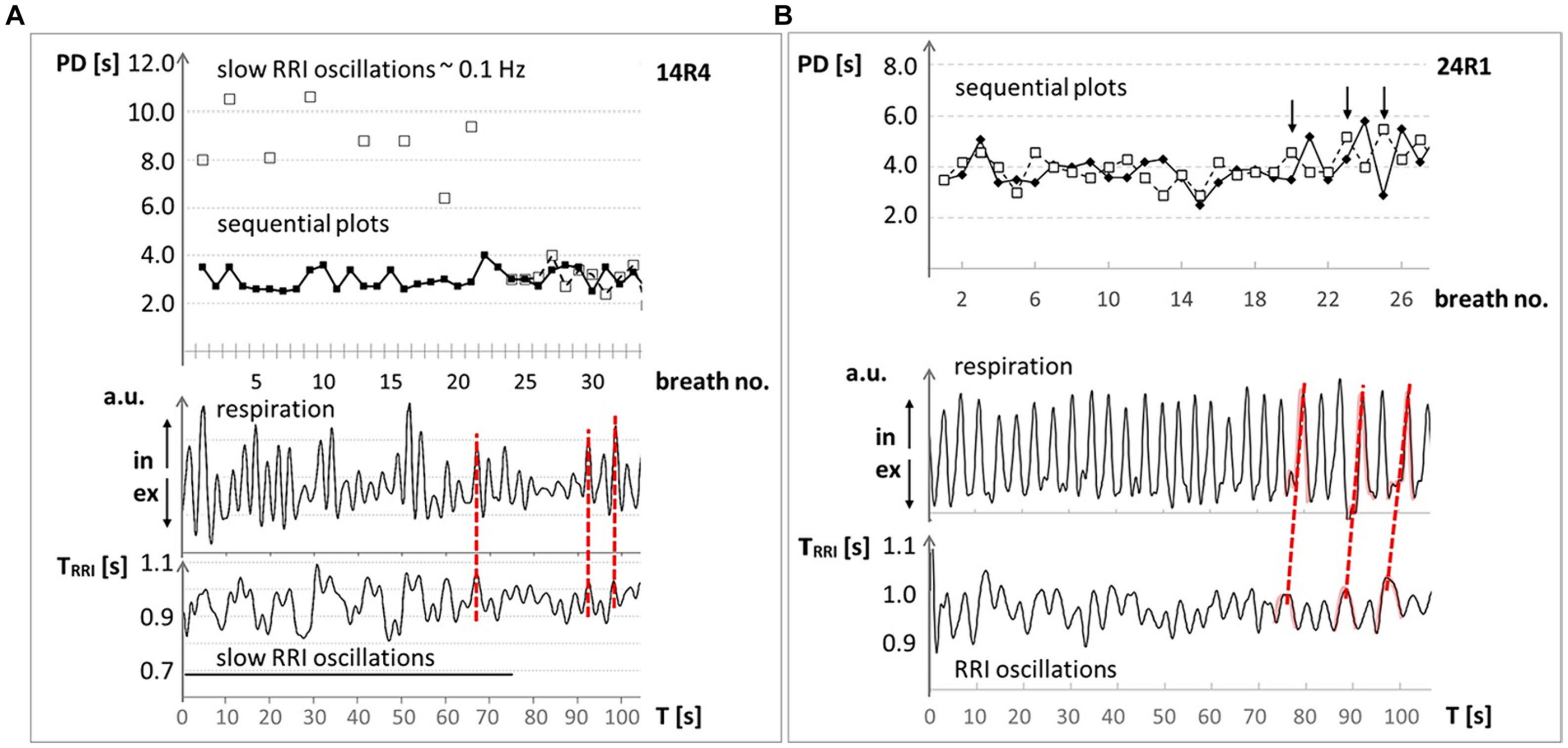
Examples of respiration and RRI time courses at ~0.32 Hz (~19 bpm) through 100 s from two MRI participants with high anxiety level. Upper panel: Sequential plot of period duration (PD) of respiratory (◆) and RRI (☐) waves, given in seconds; abscissa: breath number. Lower panel: records of respiratory (upper trace in arbitrary units (a.u.); ↑ in: inspiration, ↓ ex: expiration) and RRI waves (lower trace; RR intervals in seconds); abscissa: time in seconds. **(A)** #14: The synchronous behavior of breathing waves in both signals is marked by red broken lines and indicates a 1:3 ratio (three breaths during one RRI wave). **(B)** #24: The changes in the PD of RR intervals precede the PD of breathing waves (marked by red broken lines) indicating a dominance of the RRI rhythm over the respiratory rhythm. Modified from Rassler et al. (2022).

The dynamics of breathing waves around 0.32 Hz are best documented by a wave-by-wave analysis shown as sequential plots (Figure 1). At least two types of interactions between cardiac and breathing waves are possible, the superposition and the period duration (PD) transition (Rassler et al., 2022). In the former case, the waves of both physiological signals are phase-locked, whereby on average two, three or four breaths appear in one cardiac cycle (one RRI wave). For example, Figure 1A shows three breaths superimposed on one RRI wave in the first 60 s of the record. Figure 1B shows an example of PD transition, where the cardiac signal drives respiration. The term PD transition denotes an abrupt switch in the PD of a biological rhythm. The change in PD may comprise several cycles or even only one cycle. Concomitant or subsequent PD transitions in simultaneous rhythms occur when the rhythms are coupled. Both phenomena, superposition as well as PD transitions, indicate coupling between simultaneous rhythmic processes, which is one interesting and important way to reduce energy demand.

Respiratory sinus arrhythmia (RSA or better, positive RSA) is a typical phenomenon of the interaction between heart rate (HR) and respiration at rest. It is most dominant during slow conscious breathing with HR increase during inspiration and HR decrease during expiration (Eckberg, 1983; Hayano et al., 1996). In this case, respiration is the leading force and drives HR. Increased state anxiety promotes abnormal coupling patterns between respiration and cardiac activity. In fact, negative RSA (nRSA; Rassler et al., 2018, 2022) refers to the condition where increased state anxiety induces a complete reversal of the phase relationship between respiration and cardiac activity where the HR decreases during inspiration and increases during expiration. During nRSA, phase coupling analysis showed a reduced dominance of the respiratory rhythm over the RRI rhythm (see also Figure 1), and DTF revealed a significant information flow from RRI to respiration (Rassler et al., 2023).

## Preferred breathing rate of 0.32 Hz (19 bpm) – the binary hierarchy model of Klimesch

Klimesch’s theory (Klimesch, 2013, 2018; Rassi et al., 2019) is based on the fact that phase coupling between oscillations depends on frequencies (*f* (1), *f* (2)) with a frequency relationship r that equals an integer (*r* = f (2)/*f* (1) = integer; *f* (2) > f (1)). Thus, if different frequencies are spaced as closely as possible, r equals 2 for any frequency, relative to its closest slower integer neighbor. In this way, a binary hierarchy of frequencies emerges. This theory predicts that HR with a value of 1.25 Hz is the basic frequency for brain and other body oscillations. For delta, theta, alpha, beta, gamma 1 and gamma 2, the predicted frequencies are 2.5, 5, 10, 20, 40, and 80 Hz. For breathing, the predicted frequencies are 0.32, 0.16 and 0.08 Hz (the equivalent breathing rates are 20, 10 and 5 bpm).

The importance of HR, as basic frequency, is underlined by findings showing that mechanosensitive pyramidal neurons respond directly to heartbeat-induced pressure pulsatility (Hamill, 2023). As a consequence, heartbeat with a frequency around 1.25 Hz may be considered a sort of “pacemaker” for brain oscillations (Hamill, 2023; Klimesch, 2023) including oscillations in the respiratory centers of the brain stem (Pfurtscheller et al., 2020).

The binary hierarchy not only comprises the traditional electroencephalographic (EEG) center frequencies, but also body frequencies and slow BOLD waves as well. The center frequency of 0.32 Hz covers a range of 0.28–0.36 Hz (17–22 bpm), and the center frequency of 0.16 Hz covers a range of 0.13–0.19 Hz (see Table 1 in Klimesch, 2018). Notably, we often observed a nRSA associated with RRI oscillations at a frequency around 0.16 Hz, a dominance of the RRI rhythm over the respiratory rhythm, and a cardio-respiratory coupling ratio of 1:2.

It is important to note that frequencies of the binary hierarchy must not synchronize over prolonged time periods in a default mode or a resting situation, because this would result in a loss of the variability of the frequencies. It is assumed that synchronization occurs only task-or state-related during specific processing demands (Rodriguez-Larios and Alaerts, 2019; Soriano et al., 2023). With respect to emotional breathing, this means that BRs of 0.32 Hz or 0.16 Hz (which both belong to the binary hierarchy) only emerge if subjects face emotionally demanding situations.

## Nasal respiration at 0.32 Hz (19 bpm) entrains human limbic oscillations

The driving force of nasal respiration on neural oscillations has been known for many years (Adrian, 1942). Several studies on the rodent brain have documented oscillations in the olfactory bulb, induced by nasal breathing (Biskamp et al., 2017; Heck et al., 2019; Bagur et al., 2021; Girin et al., 2021). Tort et al. (2018) provide a timely review of the growing body of respiration-entrained brain rhythms, helping to co-ordinate the integration of distributed neural assemblies. These respiration-entrained rhythms in animals are global, but often overlooked although they are dominant in the delta and theta frequency range due to the accelerated respiration rate. Studies in patients with medically intractable epilepsy and slower respiration rhythms of ~0.16–0.32 Hz revealed nasal respiration-entrained limbic oscillations in the piriform (olfactory) cortex as well as in the amygdala and hippocampus (Zelano et al., 2016; Herrero et al., 2018). Most likely patients with high psychological stress and waiting for a surgery have a spontaneous eBR around 0.32 Hz or 19 bpm on average, only if they breathe through their nose.

**Figure 2:**
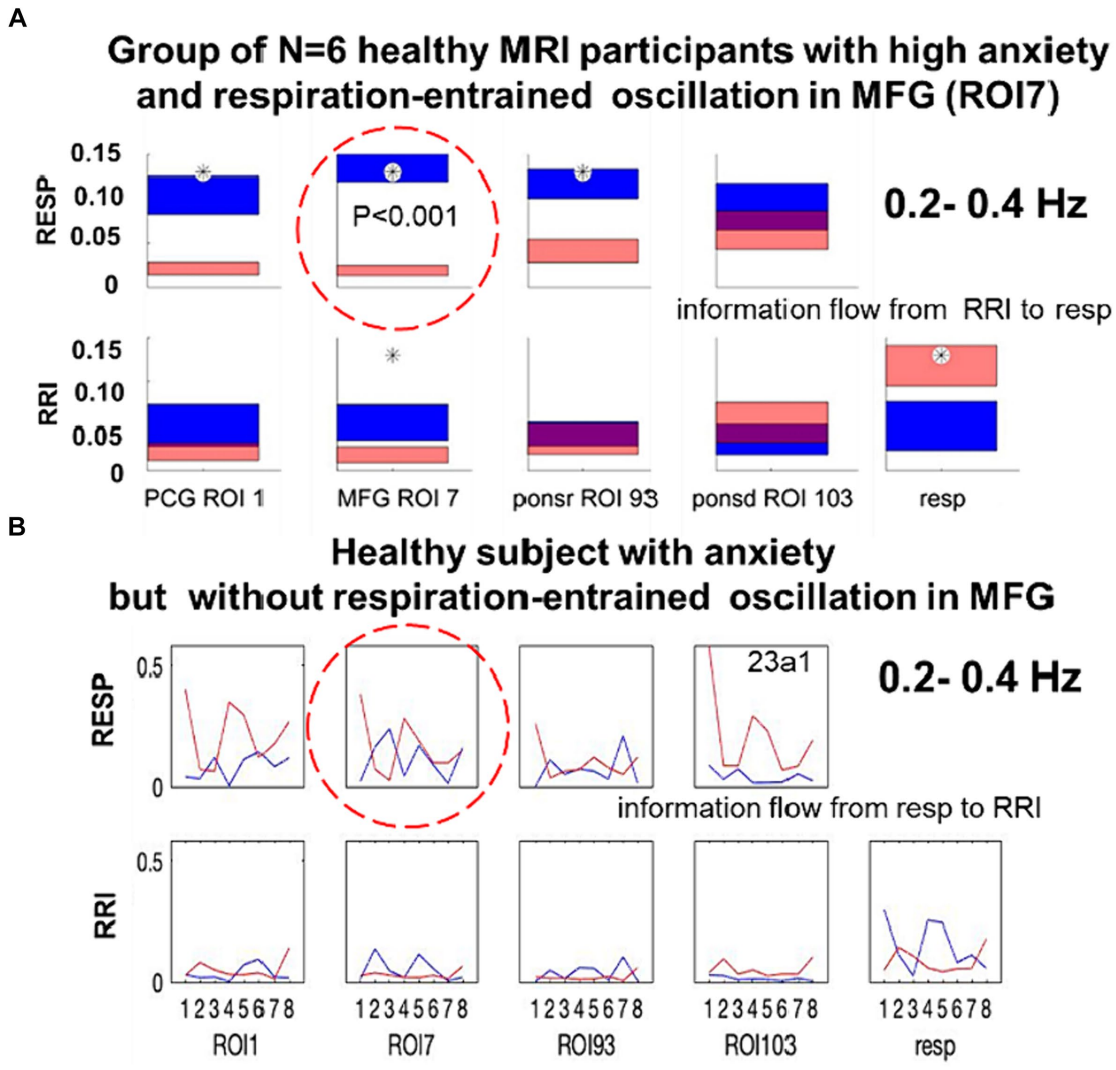
**(A)** Directed coupling strengths for the high anxiety group (*N* = 6) in the 0.2–0.4 Hz frequency band. Each box shows the strength of coupling on the vertical scale. The height of bars is proportional to the mean error. The blue color shows the flow from the signal marked below the given column to the signal marked at the left, and the red color the flow from the signal marked at left to the signal marked below. Significant differences between couplings of inflow and outflow are marked by stars (*p* < 0.05; modified from Rassler et al., 2023). **(B)** Directed coupling without respiratory-entrained oscillations in MFG from a single subject with dominant spontaneous respiration at 0.22 Hz.

In contrast to studies with rodents whose breathing frequency is close to the theta rhythm, BR in humans is much slower with a peak around 0.32 Hz, which is not always clearly documented. However, studies on air flow through the oral and nasal breathing routes confirmed that more than 80% of healthy subjects predominantly or even exclusively breathe through their nose at rest (Niinimaa et al., 1981; Amis et al., 1999). The challenging question is, how to test whether nasal breathing is dominant or not, when only BOLD signals, respiration recordings with a chest belt and electrocardiographic (ECG) recordings from the thorax are available. From iEEG data recorded from patients with epilepsy we know, that oscillatory power peaks in the prefrontal cortex (PFC) during nasal inspiration-entrained limbic oscillations. This, however, is not observed during oral breathing (Zelano et al., 2016). Notably, an enhanced number of oscillations centered at ~0.32 Hz were associated with nasal respiration-entrained human limbic oscillations. From this, one may conclude that a top-down information flow from the PFC and related areas such as, e.g., the middle frontal gyrus (MFG) to the brainstem with its respiratory and cardiovascular centers should operate in a jump-like manner like a neural avalanche in PFC and a BOLD avalanche, respectively. This “BOLD avalanche effect” during nasal respiration is documented by the significant difference (*p* < 0.001) between large downwards projections from the MFG to respiration (color blue, Figure 2A) and small upward projections from respiratory centers to the cortex (color red, Figure 2A). Note, in this case of nasal breathing, the cardiac RRI component acts as driving force for respiration in the high frequency band (Rassler et al., 2023). In the case of missing respiration-entrained oscillations in the PFC, no significant differences (no BOLD avalanche effect) between the MFG outflow and respiration inflow were found (Figure 2B). Notably, besides the strong overlap between the two flows, respiration also “acts” on the cardiac RRI signal.

## Discussion and conclusions

One basic finding is the increase in BR with a dominant peak at around 0.32 Hz (19 bpm). We assume that this elevated eBR, which is part of the binary hierarchy of brain and body oscillations, reflects increased emotional demands during a state that requires the control of anxiety (scanxiety). This elevated eBR was found in healthy persons during scanning, in patients with cancer and associated scanxiety before scanning and in patients with medically intractable epilepsy during intracranial EEG recordings. It is important to note that BR shows a broad spectrum with preferred and distinct frequencies. We assume that a state of relaxed but alert wakefulness with a breathing rate of about 0.16 Hz reflects a phenomenon that may be termed “default mode breathing (DMB).” Both preferred frequencies (0.16 and 0.32 Hz) belong to the binary hierarchy of body oscillations (Klimesch, 2018).

1. Scanxiety refers to enhanced anxiety and distress in patients with a serious disease (e.g., cancer) undergoing MRI examination. Scanxiety is observed before/ during scanning and ranked as highly concerning for patients awaiting the scan results (Derry-Vick et al., 2023). Characteristic for these patients is often an eBR of 19 bpm (~0.32 Hz). In the case of a panic reaction, a BR of 30 bpm (0.50 Hz) can be observed (Dziuda et al., 2019; Homewood and Hewis, 2023).
2. Not only in patients but also in healthy study participants, MRI-related anxiety is associated with an eBR of ~0.32 Hz (19 bpm). Most likely, every MRI participant, either healthy or patient, is confronted with some level of anxiety or in other words, anxiety-free scanning is unlikely.
3. Spontaneous nasal human breathing close to 0.32 Hz (19 bpm) entrains oscillations in the piriform (olfactory) cortex as well as in limbic-related brain structures (including amygdala and hippocampus), which are important for anxiety processing. This phenomenon was studied not only in rodents (Tort et al., 2018), but also in patients with medically intractable epilepsy and iEEG recording (Zelano et al., 2016; Herrero et al., 2018).
4. Most likely, nasal breathing at ~0.32 Hz (19 bpm) in healthy people with MRI-related anxiety also entrains oscillations in the prefrontal cortex and the limbic system (Pfurtscheller et al., 2023; Rassler et al., 2023). Strong evidence for this is the “BOLD avalanche effect”.
5. An eBR of 0.32 Hz can be considered as important physiological marker of emotional processing demands. This finding supports the binary hierarchy model, which predicts a dominant breathing frequency of 0.32 Hz and of 0.16 Hz as well. The frequency of 0.16 Hz is not only a second preferred breathing frequency but also a common central pacemaker frequency (Pfurtscheller et al., 2022).

## Author contributions

GP: Conceptualization, Writing – original draft, Writing – review & editing. BR: Writing – review & editing. GS: Writing – review & editing. WK: Writing – review & editing.

## References

[ref1] Abreu R. M. D. Cairo B. Porta A. (2023). On the significance of estimating cardiorespiratory coupling strength in sports medicine. Front Netw Physiol. 2:1114733. doi: 10.3389/fnetp.2022.1114733, PMID: 36926078 PMC10013023

[ref2] Adrian E. D. (1942). Olfactory reactions in the brain of the hedgehog. J. Physiol. 100, 459–473. doi: 10.1113/jphysiol.1942.sp003955 16991539 PMC1393326

[ref3] Amis T. C. O’Neill N. Wheatley J. R. (1999). Oral airway flow dynamics in healthy humans. J. Physiol. 515, 293–298. doi: 10.1111/j.1469-7793.1999.293ad.x, PMID: 9925899 PMC2269117

[ref4] Bagur S. Lefort M. Lacroix M. M. de Lavilléon G. Herry C. Chouvaeff M.. (2021). Breathing-driven prefrontal oscillations regulate maintenance of conditioned-fear evoked freezing independently of initiation. Nat. Commun. 12:2605. doi: 10.1038/s41467-021-22798-6, PMID: 33972521 PMC8110519

[ref5] Biskamp J. Bartos M. Sauer J. F. (2017). Organization of prefrontal network activity by respiration-related oscillations. Sci. Rep. 7:45508. doi: 10.1038/srep45508, PMID: 28349959 PMC5368652

[ref6] Bui K. T. Liang R. Kiely B. Brown C. Dhillon H. Blinman P. (2021). Scanxiety: a scoping review about scan associated anxiety. BMJ Open 11:e043215. doi: 10.1136/bmjopen-2020-043215, PMID: 34039571 PMC8160190

[ref7] Candia-Rivera D. Catrambone V. Thayer J. F. Gentili C. Valenza G. (2022). Cardiac sympathetic-vagal activity initiates a functional brain–body response to emotional arousal. PNAS 119:e2119599119. doi: 10.1073/pnas.2119599119, PMID: 35588453 PMC9173754

[ref8] Chapman H. A. Bernier D. Rusak B. (2010). MRI-related anxiety levels change within and between repeated scanning sessions. Psychiatry Res. 182, 160–164. doi: 10.1016/j.pscychresns.2010.01.005, PMID: 20409694

[ref9] Derry-Vick H. M. Heathcote L. C. Glesby N. Stribling J. Luebke M. Epstein A. S.. (2023). Scanxiety among adults with cancer: a scoping review to guide research and interventions. Cancers. 15:1381. doi: 10.3390/cancers15051381, PMID: 36900174 PMC10000102

[ref10] Dziuda Ł. Zieliński P. Baran P. Krej M. Kopka L. (2019). A study of the relationship between the level of anxiety declared by MRI patients in the STAI questionnaire and their respiratory rate acquired by a fibre-optic sensor system. Sci. Rep. 9:4341. doi: 10.1038/s41598-019-40737-w, PMID: 30867494 PMC6416391

[ref11] Ebert D. Rassler B. Hefter H. (2000). Coordination between breathing and forearm movements during sinusoidal tracking. Eur. J. Appl. Physiol. 81, 288–296. doi: 10.1007/s004210050045, PMID: 10664087

[ref12] Eckberg D. L. (1983). Human sinus arrhythmia as an index of vagal cardiac outflow. J. Appl. Physiol. 54, 961–966. doi: 10.1152/jappl.1983.54.4.961, PMID: 6853303

[ref13] Elstad M. O’Callaghan E. L. Smith A. J. Ben-Tal A. Ramchandra R. (2018). Cardiorespiratory interactions in humans and animals: rhythms for life. Am. J. Physiol. Heart Circ. Physiol. 315, H6–H17. doi: 10.1152/ajpheart.00701.2017, PMID: 29522373

[ref14] Fleming S. Thompson M. Stevens R. Heneghan C. Plüddemann A. Maconochie I.. (2011). Normal ranges of heart rate and respiratory rate in children from birth to 18 years of age: a systematic review of observational studies. Lancet 377, 1011–1018. doi: 10.1016/S0140-6736(10)62226-X, PMID: 21411136 PMC3789232

[ref15] Friedman L. Dick T. E. Jacono F. J. Loparo K. A. Yeganeh A. Fishman M.. (2012). Cardio-ventilatory coupling in young healthy resting subjects. J. Appl. Physiol. 112, 1248–1257. doi: 10.1152/japplphysiol.01424.2010, PMID: 22267392 PMC3331590

[ref16] Girin B. Juventin M. Garcia S. Lefèvre L. Amat C. Fourcaud-Trocmé N.. (2021). The deep and slow breathing characterizing rest favors brain respiratory-drive. Sci. Rep. 11:7044. doi: 10.1038/s41598-021-86525-3, PMID: 33782487 PMC8007577

[ref17] Goheen J. Anderson J. A. E. Zhang J. Norhoff G. (2023). From lung to brain – respiration modulates neural and mental activity. Neurosci. Bull. 39, 1577–1590. doi: 10.1007/s12264-023-01070-5, PMID: 37285017 PMC10533478

[ref18] Granger C. W. J. (1969). Investigating causal relations by econometric models and cross-spectral methods. Econometrica 37, 424–438. doi: 10.2307/1912791

[ref19] Hamill O. P. (2023). Pressure pulsatility links cardio-respiratory and brain rhythmicity. J. Integr. Neurosci. 22:143. doi: 10.31083/j.jin2206143, PMID: 38176935

[ref20] Harris L. M. Robinson J. Menziers R. G. (2001). Predictors of panic symptoms during magnetic resonance imaging. Int. J. Behav. Med. 8:80. doi: 10.1207/S15327558IJBM0801_06

[ref21] Hayano J. Yasuma F. Okada A. Mukai S. Fujinami T. (1996). Respiratory sinus arrhythmia. A phenomenon improving pulmonary gas exchange and circulatory efficiency. Circulation 94:842. doi: 10.1161/01.cir.94.4.842, PMID: 8772709

[ref22] Heck D. H. Kozma R. Kay L. M. (2019). The rhythm of memory: how breathing shapes memory function. J. Neurophysiol. 122, 563–571. doi: 10.1152/jn.00200.2019, PMID: 31215344 PMC6734396

[ref23] Herrero J. L. Khuvis S. Yeagle E. Cerf M. Mehta A. D. (2018). Breathing above the brain stem: volitional control and attentional modulation in humans. J. Neurophysiol. 119, 145–159. doi: 10.1152/jn.00551.2017, PMID: 28954895 PMC5866472

[ref24] Homewood H. Hewis J. (2023). “Scanxiety”: content analysis of pre-MRI patients experience on Instagram. Radiography 29, S68–S73. doi: 10.1016/j.radi.2023.01.017, PMID: 36759225

[ref25] Homma I. Masaoka Y. (2008). Breathing rhythms and emotions. Exp. Physiol. 93, 1011–1021. doi: 10.1113/expphysiol.2008.042424 18487316

[ref26] Hudson D. M. Heales C. Meertens R. (2022). Review of claustrophobia incidence in MRI: a service evaluation of current rates across a multi-Centre service. Radiography 28, 780–787. doi: 10.1016/j.radi.2022.02.010, PMID: 35279401

[ref27] Kaminski M. Blinowska K. J. (1991). A new method of the description of the information flow in the brain structures. Biol. Cybern. 65, 203–210. doi: 10.1007/BF00198091, PMID: 1912013

[ref28] Kato A. Takahashi K. Homma I. (2017). Relationship between trait and respiratory parameters during quiet breathing in normal subjects. J. Physiol. Sci. 68, 369–376. doi: 10.1007/s1256-017-0539-7 28466258 PMC5984965

[ref29] Klimesch W. (2013). An algorithm for the EEG frequency architecture of consciousness and brain body coupling. Front. Hum. Neurosci. 7:766. doi: 10.3389/fnhum.2013.00766, PMID: 24273507 PMC3824085

[ref30] Klimesch W. (2018). The frequency architecture of brain and brain body oscillations: an analysis. Eur. J. Neurosci. 48, 2431–2453. doi: 10.1111/ejn.14192, PMID: 30281858 PMC6668003

[ref31] Klimesch W. (2023). Heartbeat, brain oscillations and body awareness: a commentary. JIN. 22:155. doi: 10.31083/j.jin2206155, PMID: 38176946

[ref32] Menuet C. Connelly A. A. Bassi J. K. Melo M. R. le S. Kamar J.. (2020). PreBötzinger complex neurons drive respiratory modulation of blood pressure and heart rate. eLife 9:e57288. doi: 10.7554/eLife.57288, PMID: 32538785 PMC7326498

[ref33] Munn Z. Moola S. Lisy K. Riitano D. Murphy F. (2015). Claustrophobia in magnetic resonance imaging: a systematic review and meta-analysis. Radiography 21, e59–e63. doi: 10.1016/j.radi.2014.12.004

[ref34] Niinimaa V. Cole P. Shephard R. J. (1981). Oronasal distribution of respiratory airflow. Respir. Physiol. 43, 69–75. doi: 10.1016/0034-5687(81)90089-x, PMID: 7244427

[ref35] Pfurtscheller G. Blinowska K. J. Kaminski M. Rassler B. Klimesch W. (2022). Processing of fMRI-related anxiety and information flow between brain and body revealed a preponderance of oscillations at 0.15/0.16 Hz. Sci. Rep. 12:9117. doi: 10.1038/s41598-022-13229-7, PMID: 35650314 PMC9160010

[ref36] Pfurtscheller G. Blinowska K. J. Kaminski M. Schwerdtfeger A. R. Rassler B. Schwarz G.. (2021). Processing of fMRI-related anxiety and bi-directional information flow between prefrontal cortex and brainstem. Sci. Rep. 11:22348. doi: 10.1038/s41598-021-01710-8, PMID: 34785719 PMC8595881

[ref37] Pfurtscheller G. Kaminski M. Blinowska K. J. Rassler B. Schwarz G. Klimesch W. (2023). Respiration-entrained brain oscillations in healthy fMRI participants with high anxiety. Sci. Rep. 13:2380. doi: 10.1038/s41598-023-29482-3, PMID: 36765092 PMC9918542

[ref38] Pfurtscheller G. Schwerdtfeger A. R. Rassler B. Andrade A. Schwarz G. Klimesch W. (2020). Verification of a central pacemaker in brain stem by phase-coupling analysis between HR interval-and BOLD-oscillations in the 0.10–0.15 Hz frequency band. Front. Neurosci. 14:922. doi: 10.3389/fnins.2020.00922, PMID: 32982682 PMC7483659

[ref39] Pfurtscheller G. Schwerdtfeger A. Seither-Preisler A. Brunner C. Aigner C. Calisto J.. (2018). Synchronization of intrinsic 0.1-Hz blood-oxygen-level-dependent oscillations in amygdala and prefrontal cortex in subjects with increased state anxiety. Eur. J. Neurosci. 47, 417–426. doi: 10.1111/ejn.13845, PMID: 29368814 PMC5887876

[ref40] Rassi E. Dorffner G. Gruber W. Schabus M. Klimesch W. (2019). Coupling and decoupling between brain and body oscillations. Neurosci. Lett. 711:134401. doi: 10.1016/j.neulet.2019.134401, PMID: 31349018

[ref41] Rassler B. (2000). Mutual nervous influences between breathing and precision finger movements. Eur. J. Appl. Physiol. 81, 479–485. doi: 10.1007/s004210050071, PMID: 10774871

[ref42] Rassler B. Blinowska K. Kaminski M. Pfurtscheller G. (2023). Analysis of respiratory sinus arrhythmia and directed information flow between brain and body indicate different management strategies of fMRI-related anxiety. Biomedicines. 11:1028. doi: 10.3390/biomedicines11041028, PMID: 37189642 PMC10135951

[ref43] Rassler B. Raabe J. (2003). Co-ordination of breathing with rhythmic head and eye movements and with passive turnings of the body. Eur. J. Appl. Physiol. 90, 125–130. doi: 10.1007/s00421-003-0876-5, PMID: 12827368

[ref44] Rassler B. Schwerdtfeger A. Aigner C. S. Pfurtscheller G. (2018). "Switch-off" of respiratory sinus arrhythmia can occur in a minority of subjects during functional magnetic resonance imaging (fMRI). Front. Physiol. 9:1688. doi: 10.3389/fphys.2018.01688, PMID: 30538642 PMC6277503

[ref45] Rassler B. Schwerdtfeger A. R. Schwarz G. Pfurtscheller G. (2022). Negative respiratory sinus arrhythmia (nRSA) in the MRI-scanner – a physiologic phenomenon observed during elevated anxiety in healthy persons. Physiol. Behav. 245:113676. doi: 10.1016/j.physbeh.2021.113676, PMID: 34919919

[ref46] Rodriguez-Larios J. Alaerts K. (2019). Tracking transient changes in the neural frequency architecture: harmonic relationships between theta and alpha peaks facilitate cognitive performance. J. Neurosci. 39, 6291–6298. doi: 10.1523/JNEUROSCI.2919-18.2019, PMID: 31175211 PMC6687903

[ref47] Silvani A. Calandra-Buonaura G. Dampney R. A. Cortelli P. (2016). Brain–heart interactions: physiology and clinical implications. Philos Trans A Math Phys Eng Sci. 374:20150181. doi: 10.1098/rsta.2015.0181, PMID: 27044998

[ref48] Soriano J. R. Rodriguez-Larios J. Varon C. Castellanos N. Alaerts K. (2023). Brain-heart interactions in novice meditation practitioners during breath focus and an arithmetic task. medRxiv. doi: 10.1101/2023.07.06.23292291

[ref49] Spielberger C. D. Gorssuch R. L Lushene P. R. Vagg P. R. Jacobs G. (2009) Manual for the State-Trait Anxiety Inventory. Palo Alto, CA: Consulting Psychologists Press Inc.

[ref50] Tort A. B. Brankačk J. Draguhn A. (2018). Respiration-entrained brain rhythms are global but often overlooked. Trends Neurosci. 41, 186–197. doi: 10.1016/j.tins.2018.01.007, PMID: 29429805

[ref51] Tzeng Y. C. Larsen P. D. Galletly D. C. (2003). Cardioventilatory coupling in resting human subjects. Exp. Physiol. 88, 775–782. doi: 10.1113/eph8802606, PMID: 14603377

[ref52] Zelano C. Jiang H. Zhou G. Arora N. Schuele S. Rosenow J.. (2016). Respiration entrains human limbic oscillations and modulates cognitive function. J. Neurosci. 36, 12448–12467. doi: 10.1523/JNEUROSCI.2586-16.2016, PMID: 27927961 PMC5148230

